# Estimating Risk of Multiple Sclerosis Disease Reactivation in Pregnancy and Postpartum: The VIPRiMS Score

**DOI:** 10.3389/fneur.2021.766956

**Published:** 2022-01-17

**Authors:** Gabriel Bsteh, Harald Hegen, Katharina Riedl, Patrick Altmann, Franziska Di Pauli, Rainer Ehling, Gudrun Zulehner, Paulus Rommer, Fritz Leutmezer, Florian Deisenhammer, Thomas Berger

**Affiliations:** ^1^Department of Neurology, Medical University of Vienna, Vienna, Austria; ^2^Department of Neurology, Medical University of Innsbruck, Innsbruck, Austria; ^3^Department of Neurology, Clinic for Rehabilitation Münster, Münster, Austria

**Keywords:** multiple sclerosis, pregnancy, reactivation, activity, risk, disease modifying therapy

## Abstract

**Background:**

Evidence guiding personalized decision-making with respect to disease-modifying therapy (DMT) around pregnancy in relapsing multiple sclerosis (RMS) is lacking.

**Objective:**

To generate and validate a risk score for disease reactivation intrapartum and postpartum in RMS.

**Methods:**

From the Vienna Innsbruck MS database (VIMSD), we included 343 pregnancies in patients with RMS. Primary endpoint was disease reactivation. Patients were randomly assigned 2:1 in a generation and validation dataset. A predictive score was calculated using the Cox regression and validated.

**Results:**

In the generation dataset, occurrence of relapse and type of DMT in the year before conception, DMT washout duration, the Expanded Disability Status Scale (EDSS) at conception, and time until DMT restart postpartum were identified as independent predictors of disease reactivation (*p* < 0.001). The resulting 10-point risk score robustly predicted reactivation (explaining 75% of variance, *p* < 0.001) identifying patients at high [≥6 points; mean risk 65%; range 50–100%; hazard ratio (HR) 14.5], intermediate (3–5 points; mean risk 24%; range 15–35%; HR 4.3), and low risk (≤2 points; mean risk 6%; range 0–8%) of disease reactivation in pregnancy and up to 6 months postpartum.

**Conclusion:**

The composite Vienna Innsbruck Pregnancy Risk in Multiple Sclerosis (VIPRiMS) score is a valuable clinical tool to support patients and neurologists in anticipating risk and, thus, individualizing treatment decision-making around pregnancy.

## Introduction

Multiple sclerosis (MS), an inflammatory demyelinating disease of the central nervous system (CNS), is the most common chronic neurological disease in young adults with the risk of permanent disability (1). The incidence of MS has significantly increased in the recent decades, with women nowadays affected at least three times more often than men (2). MS predominantly affects women in reproductive age and up to 30% of women will have children after the diagnosis (3–5). The ever-broadening spectrum of disease-modifying treatments (DMTs) has enabled effective reduction of relapses and disability progression, but only very few DMTs are also approved in pregnancy (6). Thus, DMTs are usually discontinued when women wish to become pregnant or when pregnancy occurs and also because disease activity generally decreases in pregnancy (6). However, a certain relapse risk in pregnancy remains and this risk significantly increases in the first 3 months after childbirth, especially in patients who have not resumed DMT (7, 8). The decrease in relapse risk during pregnancy is most likely due to a shift from cell-mediated immunity toward humoral immunity—caused by cytokines secreted by the fetoplacental unit and estrogen. Reversal of these immunological changes after delivery leads to the increased postpartum risk for disease activity (9).

Main predictors of disease reactivation in pregnancy and postpartum are higher relapse rate in the year before (and in pregnancy) and increased disability at conception (3, 4, 8, 10). Evidence is increasing that women treated with highly effective DMT before pregnancy might be at considerable risk of disease reactivation and disability progression, especially if washout phase and time to DMT restart are prolonged (8, 11–14). Still, regulatory agencies recommend avoiding pregnancy during treatment and maintaining a 2–6-month washout period before conception (15).

The objective of this study was to generate and validate a clinical composite score for predicting disease reactivation in pregnancy and within 6 months postpartum that allows individual risk assessment and, thus, personalized treatment of patients in this period of life.

## Materials and Methods

### Patients and Definitions

For this study, we included women aged ≥18 years with relapsing MS (RMS) from the Vienna Innsbruck MS database (VIMSD), who had documented pregnancy with conception ≥12 months after MS diagnosis and who had follow-up during whole pregnancy and ≥6 months postpartum (11, 16). Pregnancies resulting in abortion, termination, or preterm delivery before the 24th gestation week were excluded to minimize confounding influence of pregnancy duration. Also, women treated with alemtuzumab, cladribine, or anti-CD20 monoclonal antibodies within ≤2 years before conception were excluded to avoid confounding by carryover DMT effects. Patients were randomly assigned 2:1 in a generation and validation dataset.

The primary endpoint was set as “clinical disease reactivation,” defined as a composite of relapse and/or disability worsening occurring between the calculated conception date and 6 months postpartum. A relapse was defined as patient-reported symptoms objectified by a neurologist or objectively observed signs typical of an acute CNS inflammatory demyelinating event with a duration of at least 24 h in the absence of fever or infection, separated from the last relapse by at least 30 days (17). Disability worsening was defined as a confirmed increase of ≥1.0 point in the Expanded Disability Status Scale (EDSS) score in patients with a baseline score of ≤ 5.5 or an increase of ≥0.5 points in patients with a baseline score of >5.5 sustained for at least 12 months as compared to baseline (18). Baseline was set as the last documented EDSS before conception.

Disease-modifying treatment was grouped as “no DMT” (N-DMT); “moderately effective DMT” (M-DMT) including interferon-beta preparations, glatiramer acetate, dimethyl fumarate, or teriflunomide; or “highly effective DMT” (H-DMT) comprising natalizumab and fingolimod. Duration of DMT washout phase was defined as the number of weeks between the last DMT application and the calculated date of conception. Time until DMT restart was defined as the number of weeks from delivery until the first DMT application postpartum.

### Statistical Analysis

The scoring system was developed and validated through the following steps (16, 19, 20):

1. In the generation dataset, the univariate Cox regressions were performed to identify those variables significantly associated with time to clinical disease reactivation. The receiver operating characteristic (ROC) analyses were used to define optimal cutoff values of the continuous variables for prediction of clinical disease reactivation. Those variables with a *p*-value less than 0.2 entered the multivariable Cox regression, where the time to clinical disease reactivation was the dependent variable. A *p*-value of 0.05 was used to select the variables to be retained in the final model. Based on the regression coefficients provided from this model, all the retained variables were allocated integral values expressing the relatively weighted impact of each variable with the overall predictive score being the sum of these values.
2. The predictive power of this score was tested by the Cox regression in the generation dataset with time to clinical disease reactivation as the dependent variable and the predictive score as the independent variable. The Kaplan–Meier survival curves were then used to calculate cumulative probabilities of clinical disease activity at 6 months postpartum for each value of the sum score.
3. In the validation dataset, the performance of the predictive score was evaluated by testing its ability in discriminating patients with low, intermediate, and high risk of clinical disease reactivation using the Kaplan–Meier survival curves and cumulative probabilities of clinical disease reactivation at 6 months postpartum. The statistical significance of intergroup heterogeneity and trend was assessed using log-rank test for trend and the Cox regression model. The goodness-of-fit models were tested by pseudo R-squared and omnibus test.

Statistical analysis was performed using the Statistical Package for the Social Sciences (SPSS) software version 26.0 (SPSS Incorporation, Chicago, Illinois, USA) and R statistical software (version 4.0.0). Missing values were handled by multiple (20 times) imputation using the missing not at random (MNAR) approach with pooling of estimates according to rules by Rubin (21). Censored data were dealt with based on the assumptions of point censoring (with interval censoring deemed unessential considering the close-meshed follow-up frequency) and independent censoring (implying that time to censoring and survival times are independent). A two-sided *p*-value < 0.05 was considered as statistically significant.

### Standard Protocol Approvals, Registrations, and Patient Consents

This study was approved by the Ethics Committee of the Medical University of Vienna (EK Nr: 2316/2020). As this was a retrospective study of anonymized data obtained in clinical routine, the requirement for informed consent was waived by decision of the Ethics Committee.

### Data Availability Statement

Anonymized data will be shared upon reasonable request from any qualified investigator after approval from the Ethics Review Board at the Medical University of Vienna.

## Results

**Figure 1:**
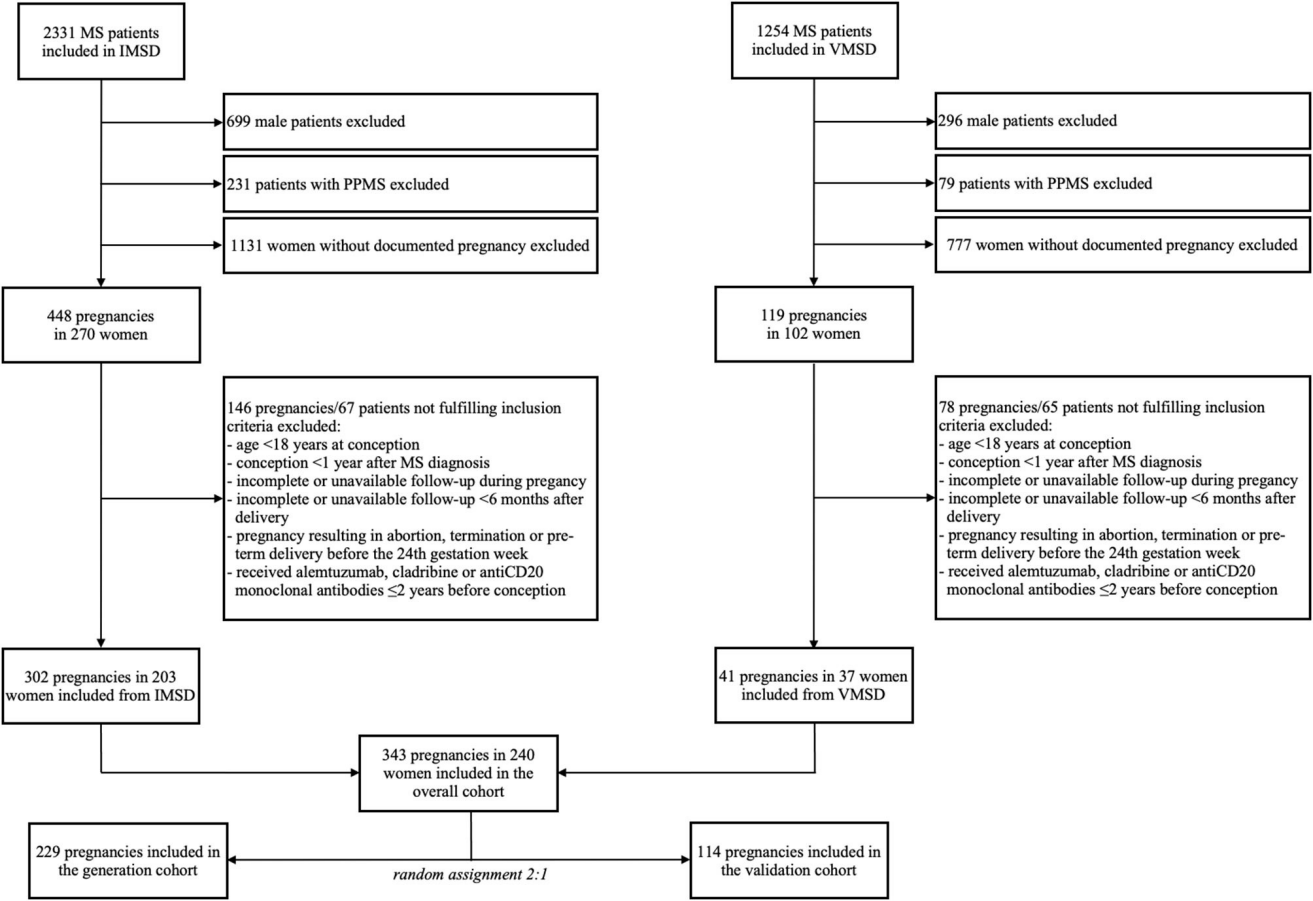
Inclusion and randomization flowchart. IMSD, Innsbruck multiple sclerosis database; MS, multiple sclerosis; PPMS, primary progressive MS; VMSD, Vienna MS database.

We included 240 female patients with RMS with 343 pregnancies. The inclusion process and random assignment to the generation and validation datasets are shown in detail in Figure 1.

Demographic and clinical characteristics of both cohorts are shown in Table 1. There were no statistically significant differences between the generation and validation cohorts. Disease reactivation occurred in 56 women (24.5%) after a median 10 months (range 1–14) in the generation cohort and in 27 women (23.7%) after a median 9 months (range 2–14) in the validation cohort.

**Table 1:** Characteristics of the generation and validation cohorts.

	**Generation cohort (*n* = 229)**	**Validation cohort (*n* = 114)**	***P*-value**
Age at disease onset^a^ (years)	26.2 (6.3)	26.4 (6.8)	0.788^d^
Age at conception^a^ (years)	30.7 (4.9)	30.4 (6.2)	0.663^d^
Disease duration at conception^a^ (years)	6.5 (5.1)	6.6 (6.2)	0.874^d^
Annualized relapse rate before conception^a^	0.38 (0.60)	0.37 (0.68)	0.889^d^
EDSS before conception^b^	1 (0–4.5)	1 (0–5.0)	0.623^e^
Number of DMTs prior to conception^b^	1 (0–3)	1 (0–3)	0.783^e^
DMT prior to conception^c^	177 (77.3)	87 (76.3)	0.840^f^
Interferon beta^c^	45 (19.7)	18 (15.8)	0.734^g^
Glatiramer acetate^c^	33 (14.4)	19 (16.7)	
Dimethyl fumarate^c^	19 (8.3)	12 (10.5)	
Fingolimod^c^	29 (12.7)	14 (12.3)	
Natalizumab^c^	51 (22.3)	24 (21.1)	
Treatment duration before conception^a^ (years)	2.4 (2.9)	2.3 (3.1)	0.760^d^
Wash out duration before conception^a^ (weeks)	5.1 (5.8)	5.3 (6.3)	0.770^d^
DMT start postpartum^c^	160 (69.9)	81 (71.1)	0.821^f^
Time until DMT restart postpartum^a^ (weeks)	19.2 (12.1)	20.1 (13.2)	0.530^d^

*DMT, disease-modifying therapy; EDSS, Expanded Disability Status Scale*.

a *mean (SD)*.

b *median (range)*.

c *absolute number (percentage)*.

d *independent t-test*.

e *Mann–Whitney U test*.

f *Fisher's exact test*.

g *Chi-squared test*.

Relapses were distributed in the generation and validation cohorts as follows: 20 (36%) and 11 (41%) in the first trimester, 7 (12%) and 3 (11%) in the second trimester, 2 (4%) and 1 (4%) in the third trimester, and 27 (48%) and 12 (44%) postpartum. A total of 50 (89.3%) and 23 (85.2%) patients had relapses only during pregnancy or postpartum, whereas 6 (10.7%) and 4 (14.8%) patients had relapses during both pregnancy as well as postpartum.

In the group of patients receiving DMT with fingolimod before conception, relapse occurred in 12/29 (7 in the first trimester, 2 in the second trimester, and 3 in the postpartum) in the generation cohort and 5/14 patients (3 in the first trimester and 2 in the postpartum) in the validation cohort. In patients on natalizumab, relapse was observed in 20/51 (11 in the first trimester, 2 in the second trimester, 2 in the third trimester, and 4 in the postpartum) and 10/24 patients (5 in the first trimester, 1 in the second trimester, 1 in the third trimester, and 3 in the postpartum).

Of the 56 and 27 respective women with disease reactivation, 7 (12%) and 4 (15%) women had more than one relapse. Six and 4 patients received H-DMT before pregnancy (natalizumab: 4 and 3 and fingolimod: 2 and 1).

**Figure 2:**
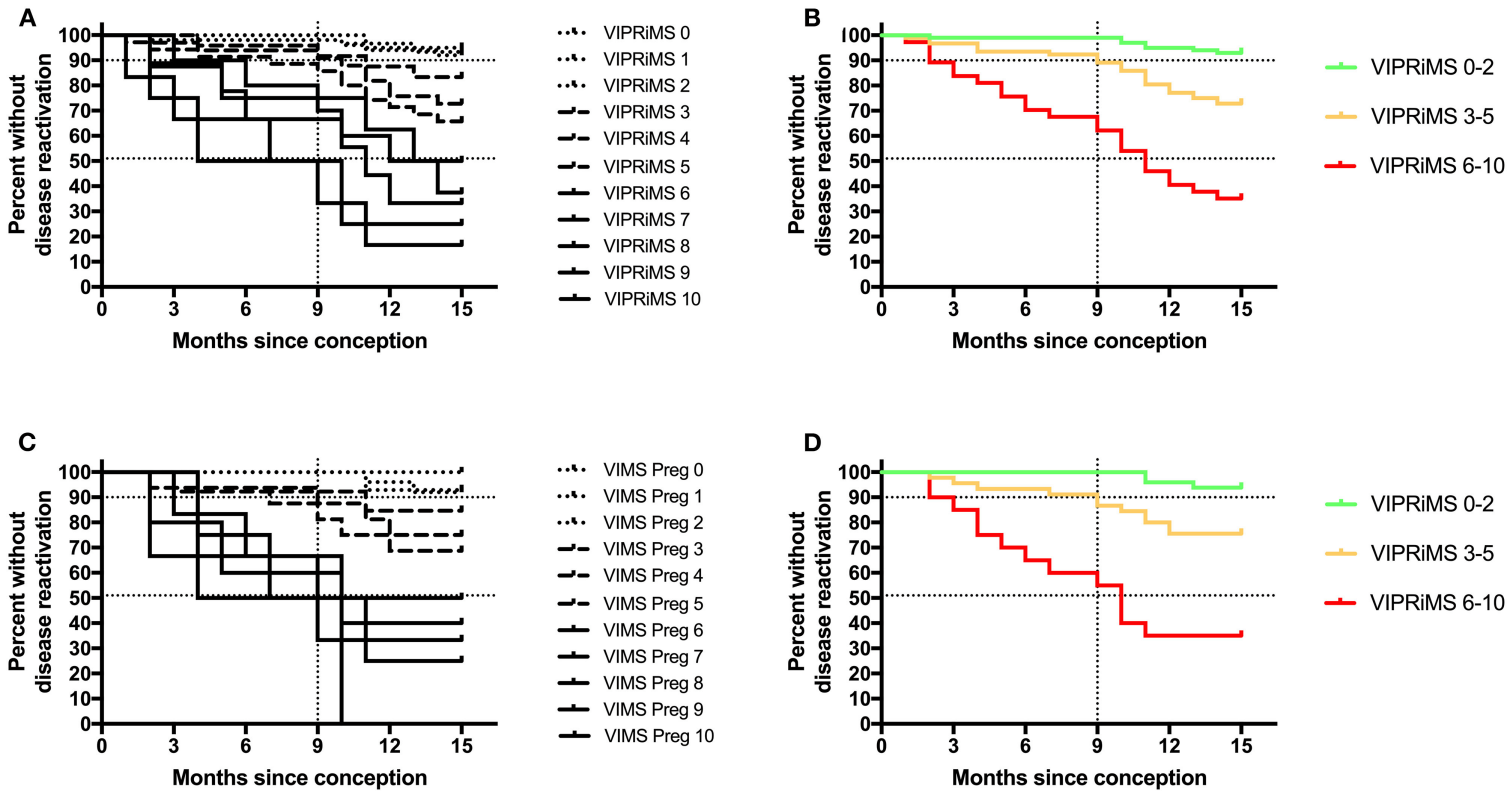
Probability of clinical disease reactivation in and after pregnancy stratified according to the Vienna Innsbruck Pregnancy Risk in Multiple Sclerosis (VIPRiMS) score in the generation cohort **(A,B)** and in the validation cohort **(C,D)**. Vertical dotted line marks the timepoint of delivery. Horizontal dotted lines indicate the 10th and 50th percentile of probability of clinical disease reactivation. Groups significantly differed in all the four graphs (*p* < 0.001, calculated by log-rank test for trend).

After analyzing the generation sample by the univariate Cox regression, five factors fulfilled criteria (*p* < 0.2) for entering the multivariable model predicting clinical disease reactivation in pregnancy or postpartum: relapse in year before conception, the EDSS before conception, DMT type before conception, duration of DMT washout phase, and time until DMT restart (*p* < 0.001). In the multivariable analysis, all the factors remained significantly associated with disease reactivation (Table 2). We assigned integral values expressing the relatively weighted impact of each variable and named the **V**ienna **I**nnsbruck **P**regnancy **R**isk **i**n **M**ultiple **S**clerosis (VIPRiMS) score. The median VIPRiMS score was 3 [interquartile range (IQR) 3, range 0–10]. The VIPRiMS score was highly predictive of clinical disease reactivation (pseudo R-squared: 0.733; omnibus *p* < 0.001) with increasing scores on the VIPRiMS correlated with increased probability of disease reactivation (Table 3; Figure 2A).

**Table 2:** Variables predicting occurrence of clinical disease reactivation in pregnancy and postpartum in the generation cohort.

**VIPRiMS**			**HR**	**95% CI**	***P*-value**	**Risk score points**
Relapse in year before conception		≥1 relapse in year before conception	3.1	1.2–4.3	<0.001	2
		<1 relapse in year before conception	Ref.			0
EDSS before conception	≥3	1.9	1.1–3.1	0.023	1	
		<3	Ref.			0
DMT type before conception		Highly-effective DMT (H-DMT)	4.3	2.5–7.1	<0.001	3
		Moderately-effective DMT (M-DMT)	1.8	0.8–3.5	0.097	0
		No DMT	Ref.			0
Duration of DMT wash-out phase	H-DMT	>12 weeks	3.2	2.1–4.3	<0.001	2
		4–12 weeks	2.3	1.4–4.1	<0.001	1
		<4 weeks	Ref.			0
	M-DMT	>12 weeks	2.0	1.2–3.7	0.004	1
		≤ 12 weeks	Ref			0
	N-DMT	NA				0
Time until DMT restart postpartum	H-DMT	>8 weeks	3.3	1.7–7.4	<0.001	2
		4–8 weeks	2.1	1.4–4.4	<0.001	1
		<4 weeks	Ref			0
	M-DMT	>12 weeks	2.2	1.5–4.8	<0.001	1
		≤ 12 weeks	Ref			0
	N-DMT	>12 weeks	1.9	1.2–3.4	0.011	1
		≤ 12 weeks	Ref			0

*DMT, disease-modifying therapy; EDSS, Expanded Disability Status Scale; H-DMT, highly effective DMT comprising natalizumab and fingolimod; M-DMT, moderately effective DMT including interferon-beta preparations, glatiramer acetate, dimethyl fumarate, or teriflunomide; N-DMT, no DMT; HR, hazard ratio; Ref., reference category. Calculated by the multivariate Cox regression model (pseudo R-squared: 0.733; omnibus p < 0.001)*.

**Table 3:** Probability of clinical disease activation in pregnancy and within 6 months postpartum stratified according to the Vienna Innsbruck Pregnancy Risk in Multiple Sclerosis (VIPRiMS) score.

**VIPRiMS**		**Generation cohort**			**Validation cohort**		
		Patients at risk	Patients with disease reactivation	Probability of disease reactivation^a^	Patients at risk	Patients with disease reactivation	Probability of disease reactivation^a^
0	Low risk	20	1	5.0	10	0	0.0
1		30	2	6.6	14	1	7.1
2		50	4	8.0	25	2	8.0
3	Intermediate risk	24	4	16.7	13	2	15.3
4		33	9	27.3	16	4	25.0
5		35	12	34.3	16	5	31.3
6	High risk	10	5	50.0	6	3	50.0
7		8	5	62.5	4	3	75.0
8		9	6	66.6	5	3	60.0
9		6	5	83.3	3	2	66.6
10		4	3	75.0	2	2	100.0
Total		229	56	24.5	114	27	23.7

a *percentage, cumulatively calculated at 6 months postpartum*.

Then, we grouped patients according to probability of disease reactivation in the generation dataset as subjects with low risk (i.e., below the 10th percentile, the VIPRiMS score 0–2), intermediate (i.e., between 10 and 50th percentile, the VIPRiMS score 3–5), and high risk (i.e., above the 50th percentile, the VIPRiMS score 6–10) of disease reactivation. The probability of clinical disease reactivation within 15 months after conception, i.e., at 6 months postpartum, was 7.0% in the low-risk group, 27.2% in the intermediate-risk group, and 64.9% in the high-risk group (*p* < 0.001, Figure 2B). Taking the low-risk group as reference, hazard ratios (HRs) were 4.3 (95% CI: 1.9–10.0; *p* = 0.001) for the intermediate-risk group and 14.5 (95% CI: 6.2–33.7; *p* < 0.001) for the high-risk group.

In the validation cohort, the median VIPRiMS score was 3 (IQR 3, range 0–10), which did not significantly differ from the generation cohort. The VIPRiMS score was also strongly predictive of disease reactivation as demonstrated in the Cox regression model (pseudo R-squared: 0.749; omnibus *p* < 0.001). The low-risk group displayed a 6.1% probability of disease reactivation within 15 months after conception, which was significantly lower compared to 24.4% in the intermediate-risk group and 65.0% in the high-risk group (Figures 2C,D).

## Discussion

In clinical routine, counseling women with MS planning pregnancy on their risk of relapse and disability progression, when DMT should be discontinued and when DMT should be (re)started postpartum remains challenging. As commonly as these questions arise, there is still little evidence-based guidance for neurologists counseling patients. While the majority of women probably do not need DMT in pregnancy, women with highly active MS require a more differentiated approach. In this study, we aimed to generate and validate a clinical composite score for predicting disease reactivation in individual women with RMS in case of pregnancy.

The multivariable analysis in the generation sample revealed five factors independently predictive of disease reactivation in pregnancy and postpartum: (a) occurrence of relapse in the year before conception (3-fold increased risk), (b) EDSS ≥3 at conception (2-fold increased risk), (c) treatment with highly-effective DMT before conception (4-fold increased risk), (d) DMT washout time before conception (>2-fold increased risk if washout time >4 weeks for H-DMT and >12 weeks for M-DMT), and (e) time until DMT restart postpartum (>2-fold increased risk if >4 weeks for H-DMT and >12 weeks for M-DMT).

Based on this model, a score combining these factors (VIPRiMS) was generated, which was able to stratify patients at low (VIPRiMS ≤ 2), intermediate (VIPRiMS 3–5), and high risk (VIPRiMS ≥6) of disease reactivation with probabilities of 7, 27, and 65%, respectively. The VIPRiMS score was reliably attributable to the validation sample with disease reactivation probabilities of 6, 24, and 65% for the low-, intermediate-, and high-risk groups. Compared to the low-risk group, the intermediate-risk group displayed a 4-fold increased risk of disease reactivation, while the high-risk group displayed a 14-fold increased risk of disease reactivation.

The reliability of the VIPRiMS score is underlined by high goodness-of-fit parameters indicating that about 75% of the variation in the risk of disease reactivation in pregnancy and postpartum is explained by the VIPRiMS.

Our results are in line with earlier studies, where risk of relapse and disability progression in pregnancy and postpartum is predicted by preconception relapse activity and the higher EDSS at conception, but also by application of H-DMT preconception and prolonged periods of DMT washout and postpartum to DMT restart (3, 10–13). Demographic characteristics, age at conception, disease duration, preconception disease activity, and disability as well as the rate of disease reactivation (24.5% in the generation cohort and 23.7% in the validation cohort) are well within the range reported in the literature (7, 10, 22). This study extends the impact of these factors by combining them into an easily applicable risk score.

Managing women with MS of childbearing potential requires an individual strategy covering pregnancy planning, pregnancy, and the postpartum period. Counseling should cover DMT safety in all the three phases. Disease activity, type and impact of DMT withdrawal, and potential DMT effects on the fetus should all be considered in DMT decisions (15). Since disease activity typically decreases in pregnancy, most women are able to safely discontinue treatment for pregnancy. However, in women with highly active MS or on H-DMT with risk of disease reactivation upon discontinuation treatment should be planned carefully before pregnancy to decrease relapse risk. In this group, preconception untreated intervals should be kept as short as possible and can be bridged with other DMTs, which can be extended until conception or even in pregnancy and DMT should be restarted postpartum early (15). Obviously, these decisions need to be evaluated and discussed individually in every case. The VIPRiMS score can aid in estimating and quantifying the implications of various strategies considered.

### Strengths and Limitations

The main strengths of this study are its population-based approach and the detailed characterization of the study cohort provided by the high-quality data from certified specialized MS centers. The characteristics of the study cohorts with respect to age at conception, disease duration, and disease activity are in line with other cohorts presented in the literature suggesting generalizability of our results (3, 4, 7, 10, 23). Another strength is the robust and standardized statistical approach to generation and validation of the risk score (16, 19, 20).

As a limitation, we did not include MRI results in this study because they were not obtained systematically and used varying protocols. MRI might provide additional information in risk stratification for relapse and disability progression in pregnancy or postpartum lacking in this study (24). Also, it needs to be stressed that women treated with alemtuzumab, cladribine, or anti-CD20 monoclonal antibodies within ≤ 2 years before conception were excluded. Hence, the VIPRiMS score cannot be extrapolated to women treated with these DMTs. This is an important future direction. Also, our cohort did not include patients receiving DMT during pregnancy, which is currently applied in certain risk constellations, e.g., with women on natalizumab. As our cohort includes 240 pregnancies with first childbirth and 143 pregnancies with subsequent childbirths, we conducted a sensitivity analyses leaving out the subsequent childbirths and did not detect any significant change of results. We could not adjust for the effect of breastfeeding, since we did not have sufficient data. The relationship between breastfeeding and postpartum relapse is controversially discussed with some studies reporting exclusive breastfeeding to decrease risk of disease reactivation, while others did not (11, 25–27). This might be explained by a bias based on different disease activity, i.e., women with low disease activity are less likely to restart DMT immediately after delivery and, therefore, more likely to breastfeed. Still, we cannot definitely rule out a potential confounding effect of breastfeeding in this study. However, in women with high-active MS, breastfeeding should not delay reinitiating DMT, especially since evidence is growing that monoclonal antibodies and injectable DMTs can be safely applied concomitantly to breastfeeding (4, 15, 28). In women with low risk of disease reactivation, exclusive breastfeeding for 3–6 months might be encouraged, if possible (15). Also, it has to be acknowledged that this study cohort stems from two centers with similar general treatment strategies for counseling patients with respect to MS and pregnancy, which may be a potential source of bias, which we cannot formally account for. Therefore, the VIPRiMS score requires external validation in an independent cohort. Finally, we did not include women with pregnancies resulting in abortion, termination, or preterm delivery before the 24th gestation week. There is evidence that loss of pregnancy may be associated with short-term disease reactivation (29, 30). However, the VIPRiMS score is currently not applicable to these patients as well.

## Conclusion

In conclusion, the VIPRiMS score is an easy and practicable tool to estimate the risk of clinical disease activity in RMS informing patients and neurologists in planning pregnancy and individually tailoring decisions, if and when, to discontinue DMTs.

## Data Availability Statement

The raw data supporting the conclusions of this article will be made available by the authors, without undue reservation.

## Ethics Statement

The studies involving human participants were reviewed and approved by Ethics Committee of the Medical University of Vienna (EK Nr: 2316/2020). Written informed consent for participation was not required for this study in accordance with the national legislation and the institutional requirements.

## VIMSD Investigators in Alphabetical Order

Altmann, Patrick (Medical University of Vienna, Vienna, Austria); Auer, Michael (Medical University of Innsbruck, Innsbruck, Austria); Berek, Klaus (Medical University of Innsbruck, Innsbruck, Austria); Berger, Thomas (Medical University of Vienna, Vienna, Austria); Bsteh, Gabriel (Medical University of Vienna, Vienna, Austria); Deisenhammer, Florian (Medical University of Innsbruck, Innsbruck, Austria); Di Pauli, Franziska (Medical University of Innsbruck, Innsbruck, Austria); Ehling, Rainer (Department of Neurology, Clinic for Rehabilitation Münster, Münster, Austria); Hegen, Harald (Medical University of Innsbruck, Innsbruck, Austria); Kornek, Barbara (Medical University of Vienna, Vienna, Austria); Leutmezer, Fritz (Medical University of Vienna, Vienna, Austria); Monschein, Tobias (Medical University of Vienna, Vienna, Austria); Rinner, Walter (Medical University of Vienna, Vienna, Austria); Rommer, Paulus (Medical University of Vienna, Vienna, Austria); Schmied, Christiane (Medical University of Vienna, Vienna, Austria); Wurth, Sebastian (Medical University of Graz, Graz, Austria); Zebenholzer, Karin (Medical University of Vienna, Vienna, Austria); Zinganell, Anne (Medical University of Innsbruck, Innsbruck, Austria); Zulehner, Gudrun (Medical University of Vienna, Vienna, Austria); Zrzavy, Tobias (Medical University of Vienna, Vienna, Austria).

## Author Contributions

GB had full access to all the data in this study and takes responsibility for the integrity of the data and the accuracy of the data analysis. GB and HH contribute to the study concept and design, patient recruitment, acquisition of data, statistical analysis and interpretation of data, and drafting of manuscript. KR, PA, FDP, RE, GZ, PR, FL, and FD contribute to the patient recruitment, acquisition of data, and critical revision of manuscript for intellectual content. TB contributes to the study concept and design, patient recruitment, interpretation of data, critical revision of manuscript for intellectual content, and study supervision. All authors contributed to the article and approved the submitted version.

## Conflict of Interest

GB has participated in meetings sponsored by, received speaker honoraria, or travel funding from Biogen, Celgene, Lilly, Merck, Novartis, Roche, Sanofi Genzyme, and Teva and received honoraria for consulting Biogen, Celgene, Roche, and Teva. HH has participated in meetings sponsored by, received speaker honoraria, or travel funding from Bayer, Biogen, Merck, Novartis, Sanofi Genzyme, Siemens, and Teva and received honoraria for consulting Biogen, Celgene, Novartis, and Teva. PA has participated in meetings sponsored by, received speaker honoraria, or travel funding from Biogen, Merck, Roche, Sanofi Genzyme, and Teva and received honoraria for consulting from Biogen. He received a research grant from Quanterix International and was awarded a combined sponsorship from Biogen, Merck, Roche, Sanofi Genzyme, and Teva for a clinical study. FDP has participated in meetings sponsored by, received honoraria (lectures, advisory boards, and consultations), or travel funding from Bayer, Biogen, Celgene, Merck, Novartis, Roche, Sanofi Genzyme, and Teva. RE has participated in meetings sponsored by, received speaker honoraria, or travel funding from Almirall, Biogen, Böhringer Ingelheim, Celgene, Daiichi Sankyo, Merck, Novartis, Ottobock, and Teva. GZ has participated in meetings sponsored by or received travel funding from Biogen, Merck, Novartis, Roche, Sanofi Genzyme, and Teva. PR has received honoraria for consultancy/speaking from AbbVie, Alexion, Almirall, Biogen, Merck, Novartis, Roche, Sandoz, and Sanofi Genzyme and has received research grants from Amicus, Biogen, Merck, and Roche. FL has participated in meetings sponsored by or received honoraria for acting as an advisor/speaker for Actelion, Alexion, Almirall, Bayer, Biogen, Celgene, Medday, Merck, Novartis, Octapharma, Pfizer, Roche, Sanofi-Aventis, and Teva. FD has participated in meetings sponsored by or received honoraria for acting as an advisor/speaker for Alexion, Almirall, Biogen, Celgene, Merck, Novartis, Roche, and Sanofi Genzyme. His institution received scientific grants from Biogen and Sanofi Genzyme. TB has participated in meetings sponsored by and received honoraria (lectures, advisory boards, and consultations) from pharmaceutical companies marketing treatments for MS: Allergan, Almirall, Bayer, Biogen, Biologix, Bionorica, Celgene/Bristol Myers Squibb (BMS), GlaxoSmithKline (GSK), Janssen-Cilag, Medday, Merck, Novartis, Octapharma, Roche, Sanofi Genzyme, Teva, and TG Pharmaceuticals. His institution has received financial support in the past 12 months by unrestricted research grants Biogen, Celgene/BMS, Merck, Novartis, Sanofi Genzyme, and Teva and for participation in clinical trials in MS sponsored by Alexion, Biogen, Celgene/BMS, Merck, Novartis, Octapharma, Roche, Sanofi Genzyme, and Teva. The remaining author declares that the research was conducted in the absence of any commercial or financial relationships that could be construed as a potential conflict of interest.

## Publisher's Note

All claims expressed in this article are solely those of the authors and do not necessarily represent those of their affiliated organizations, or those of the publisher, the editors and the reviewers. Any product that may be evaluated in this article, or claim that may be made by its manufacturer, is not guaranteed or endorsed by the publisher.
